# Coherent Magnons with Giant Nonreciprocity at Nanoscale Wavelengths

**DOI:** 10.1021/acsnano.3c08390

**Published:** 2024-02-05

**Authors:** Rodolfo Gallardo, Markus Weigand, Katrin Schultheiss, Attila Kakay, Roland Mattheis, Jörg Raabe, Gisela Schütz, Alina Deac, Jürgen Lindner, Sebastian Wintz

**Affiliations:** † Universidad Técnica Federico Santa María, 2390123 Valparaíso, Chile; ‡ Helmholtz-Zentrum Berlin, 12489 Berlin, Germany; § Helmholtz-Zentrum Dresden-Rossendorf, Insitute of Ion Beam Physics and Materials Research, 01328 Dresden, Germany; ∥ Leibniz Institut für Photonische Technologien, 07745 Jena, Germany; ⊥ Paul Scherrer Institut, 5232 Villigen PSI, Switzerland; # Max-Planck-Institut für Intelligente Systeme, 70569 Stuttgart, Germany; ▼ Helmholtz-Zentrum Dresden-Rossendorf, Dresden High Magnetic Field Laboratory, 01328 Dresden, Germany

**Keywords:** spin waves, magnons, nonreciprocity, caustics, X-ray microscopy

## Abstract

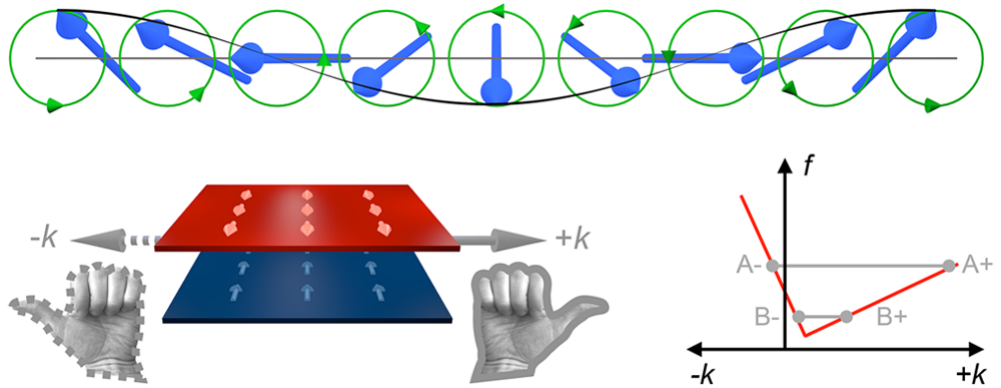

Nonreciprocal wave propagation arises in systems with broken time-reversal symmetry and is key to the functionality of devices, such as isolators or circulators, in microwave, photonic, and acoustic applications. In magnetic systems, collective wave excitations known as magnon quasiparticles have so far yielded moderate nonreciprocities, mainly observed by means of incoherent thermal magnon spectra, while their occurrence as coherent spin waves (magnon ensembles with identical phase) is yet to be demonstrated. Here, we report the direct observation of strongly nonreciprocal propagating coherent spin waves in a patterned element of a ferromagnetic bilayer stack with antiparallel magnetic orientations. We use time-resolved scanning transmission X-ray microscopy (TR-STXM) to directly image the layer-collective dynamics of spin waves with wavelengths ranging from 5 μm down to 100 nm emergent at frequencies between 500 MHz and 5 GHz. The experimentally observed nonreciprocity factor of these counter-propagating waves is greater than 10 with respect to both group velocities and specific wavelengths. Our experimental findings are supported by the results from an analytic theory, and their peculiarities are further discussed in terms of caustic spin-wave focusing.

Nonreciprocity is a fundamental phenomenon in systems hosting propagating waves and refers to the difference in a certain wave quantity (amplitude, frequency, wavelength, etc.) for counter-propagating waves. In general, it is a consequence of time-reversal symmetry breaking due to various possible origins.^1^ This symmetry breaking may stem from an external bias (such as a magnetic field) in linear systems or could be a result of self-biasing in nonlinear systems. Nonreciprocity was found in basic works, e.g., for the case of electromagnetic radiation in the microwave^1−3^ and optical regime^4−7^ as well as for sound waves in fluids and solids.^8^ At the same time, wave nonreciprocity is key to the operation of isolator and circulator devices, where the first allows signal transfer only in one direction between two ports and the second transmits any signal from port to port in a defined rotation sense of a three-terminal scheme. Such devices are commonly used in microwave-, photonic-, and acoustic technologies, with—among many others—the prominent example of signal duplexing in radar operation.

Spin waves, as the fundamental excitations of ordered spin systems,^9^ represent another instance of waves that can exhibit nonreciprocity. In recent years, spin waves have been proposed as signal carriers for future spintronic logic devices. The corresponding field of research is termed magnonics, named after the magnon as the quantum of spin-wave excitation. A potential advantage of harnessing spin waves, instead of the electric charges utilized in present microelectronics, is the prevention of Ohmic losses in the signal transfer, enabling a lower power consumption in operation. Likewise, the several orders of magnitude shorter wavelengths of magnons compared to electromagnetic waves of the same frequency could allow for an additional miniaturization of signal processing circuits.^10,11^ This could apply to conventional logic or signal processing devices but also to unconventional schemes such as wave-based analog processors or neuromorphic computing, with the latter exploiting intrinsic spin-wave nonlinearities.^12,13^ Typically, the wavelengths of spin waves range from millimeters to sub-nanometers, and their frequencies extend from the MHz into the THz domain.^14^

In a classical view, a coherent spin wave can be seen as a set of dynamically precessing magnetic moments (at frequency *f*) exhibiting a spatial phase shift that defines the wavelength λ and its inverse wavenumber *k* = 2π/λ; see Figure 1(a). The functional relation between the wavenumber and the frequency of a spin wave is termed the spin-wave dispersion relation *f*(*k*), giving rise to the phase velocity *v*_ph_ = 2π*f*/*k* and the group velocity *v*_g_ = 2π(d*f*/d*k*). The most relevant energies determining such dispersion relations are the magnetic dipolar energy (dominant for long wavelengths, referred to as magnetostatic waves) and the exchange energy (dominant for short wavelengths, referred to as exchange waves), together with magnetic anisotropy energy.^14^ In thin-film systems with in-plane magnetization (**M**), spin waves exhibit strongly anisotropic properties with respect to their two basic propagation geometries: perpendicular to **M** [Damon–Eshbach (**k** = *k***e**_k_ ⊥ **M**)] and collinear to **M** [Backward-Volume (**k** || **M**)], respectively.^14^

**Figure 1:**
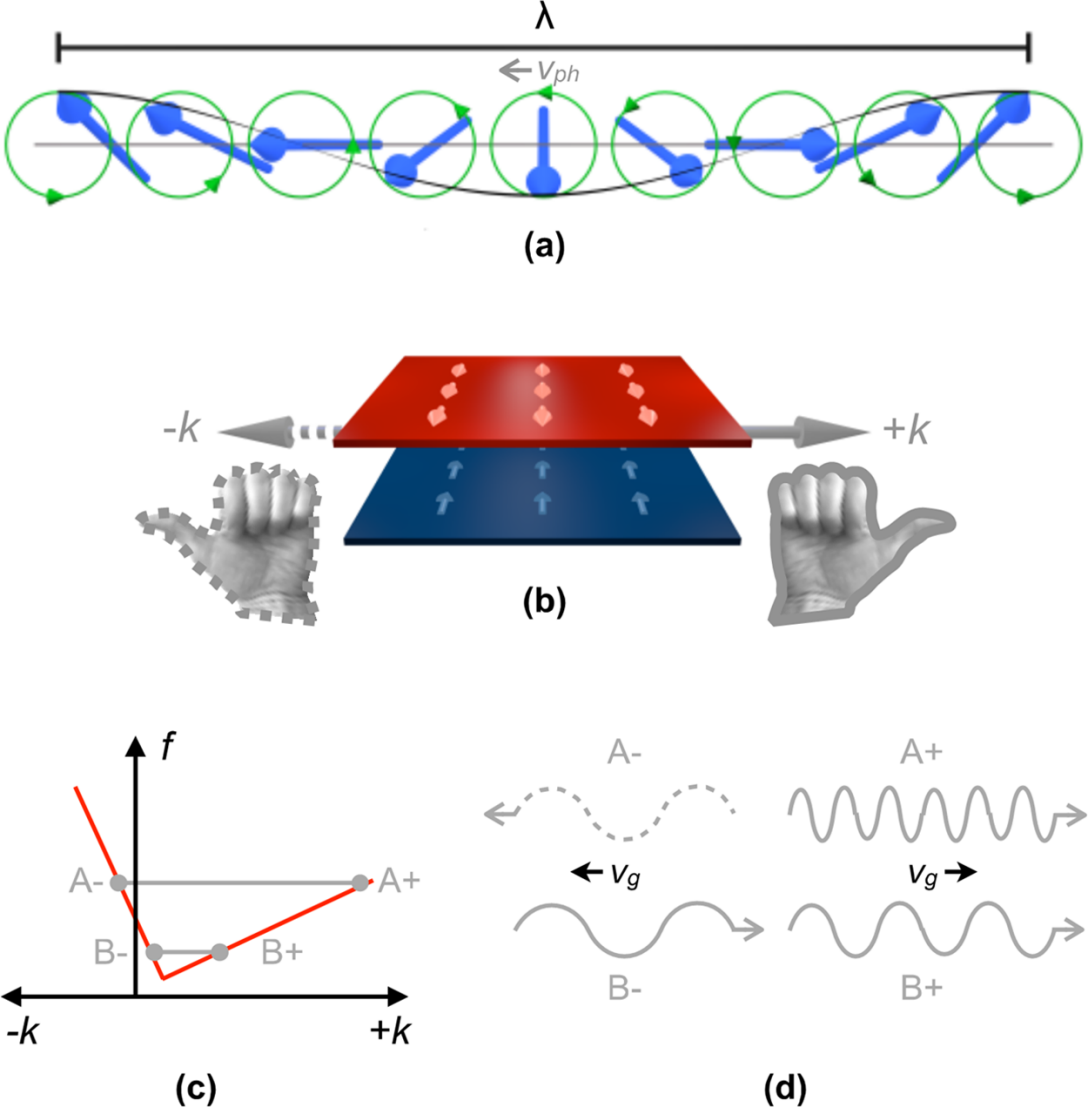
Schematic magnon nonreciprocity. (a) A spin wave: Magnetic moments (blue arrows) precess along the green trajectories, the locations of their tips forming a wave of wavelength λ. (b) Chirality of an antiparallel magnetic bilayer (magnetizations indicated by arrows) with respect to magnon phase propagation along +*k* (right-handed) and −*k* (left-handed). (c) Simplified magnon dispersion schematic *f*(*k*) (red line) in an antiparallel magnetic bilayer with anisotropy. (d) Spin waves at characteristic dispersion points (A+, A–, B+, B−) with phase velocities indicated by gray arrows and group velocities indicated by black arrows, respectively.

Spin-wave nonreciprocity^15^ may occur with respect to amplitude, as it is most pronounced for classical Damon–Eshbach surface waves,^16^ yet to a lesser extent also for their thin film counterparts of the same geometry.^17,18^ In both cases, the amplitude decays exponentially over the crystal/film thickness as a function of *k*, with the sense of decay (top vs bottom surface) depending on the spin-wave propagation direction. This is in contrast to backward volume modes that do not show any nonreciprocity in ideal systems. Furthermore, it was found that counter-propagating waves of the same frequency *f*_0_ exhibit different wavelengths/wavenumbers^19,20^ [*f*_0_ (*k*^*+*^) = *f*_0_ (*k*^*-*^), but *k*^*+*^ ≠ |*k*^*-*^*|*] for systems exhibiting a chiral Dzyaloshinskii–Moriya interaction.^21−23^ Note that spin-wave nonreciprocity is fundamentally different from spin-wave excitation asymmetries that may result from the antenna geometry.^24^

As predicted by Grünberg in 1981,^25^ such dispersion nonreciprocity may even be stronger in magnetic bilayers with antiparallel orientation of magnetizations (**M**_1_ ↑↓ **M**_2_) for waves of the layer-collective eigenmode in the Damon–Eshbach geometry (**k** ⊥ **M**_i_).^15,26−37^ Here, chirality is given by the circulation of magnetizations **M**_i_ with respect to the wave propagation direction ± **k**, see Figure 1(b). Magnetic anisotropy, in addition, causes the frequency minimum of the spin-wave dispersion relation to be displaced on the *k*-axis; see Figure 1(c).^33,16^ In such a situation, two different nonreciprocal regimes of spin-wave propagation can be identified [see Figure 1(d)]: At low isofrequency dispersion points (B^i^), both waves have positive phase velocities, and while for higher positive *k* the group velocity is positive (B^+^), it is negative for smaller positive *k* (B^–^). At higher isofrequency dispersion points (A^i^), positive phase velocities always coincide with positive group velocities and *vice versa* at a remaining *k-*nonreciprocity.

So far, however, experimental demonstrations of such spin-wave nonreciprocity effects in antiparallel bilayers are of moderate magnitude, with nonreciprocity factors (the ratio of a specific wave quantity for counter-propagating waves) not exceeding five and typically being below two. Furthermore, existing studies mainly rely on scattering experiments of incoherent, thermally excited magnons with wavelengths at or above ∼250 nm, as a result of the detection limits of optical techniques.^27,30,31,34,35^ Here, we report the direct phase-resolved imaging of coherent nonreciprocal spin waves in a magnetic bilayer with antiparallel magnetizations using magnetic X-ray microscopy. Thereby, we demonstrate an intrinsic, large nonreciprocity occurring at nano- and microscale wavelengths in this system. In particular, we observe spin waves from sub-100 nm to 5 μm wavelengths at frequencies up to 5 GHz with a nonreciprocity factor in the linearized group velocity (*v̅*_g^–^_/*v̅*_g^+^_) of more than 10 without any magnetic bias field. We support our findings with an analytic theory, and we outline broadband caustic self-focusing of spin waves based on nonreciprocity in terms of magnonic applications.^38^

## Results and Discussion

**Figure 2:**
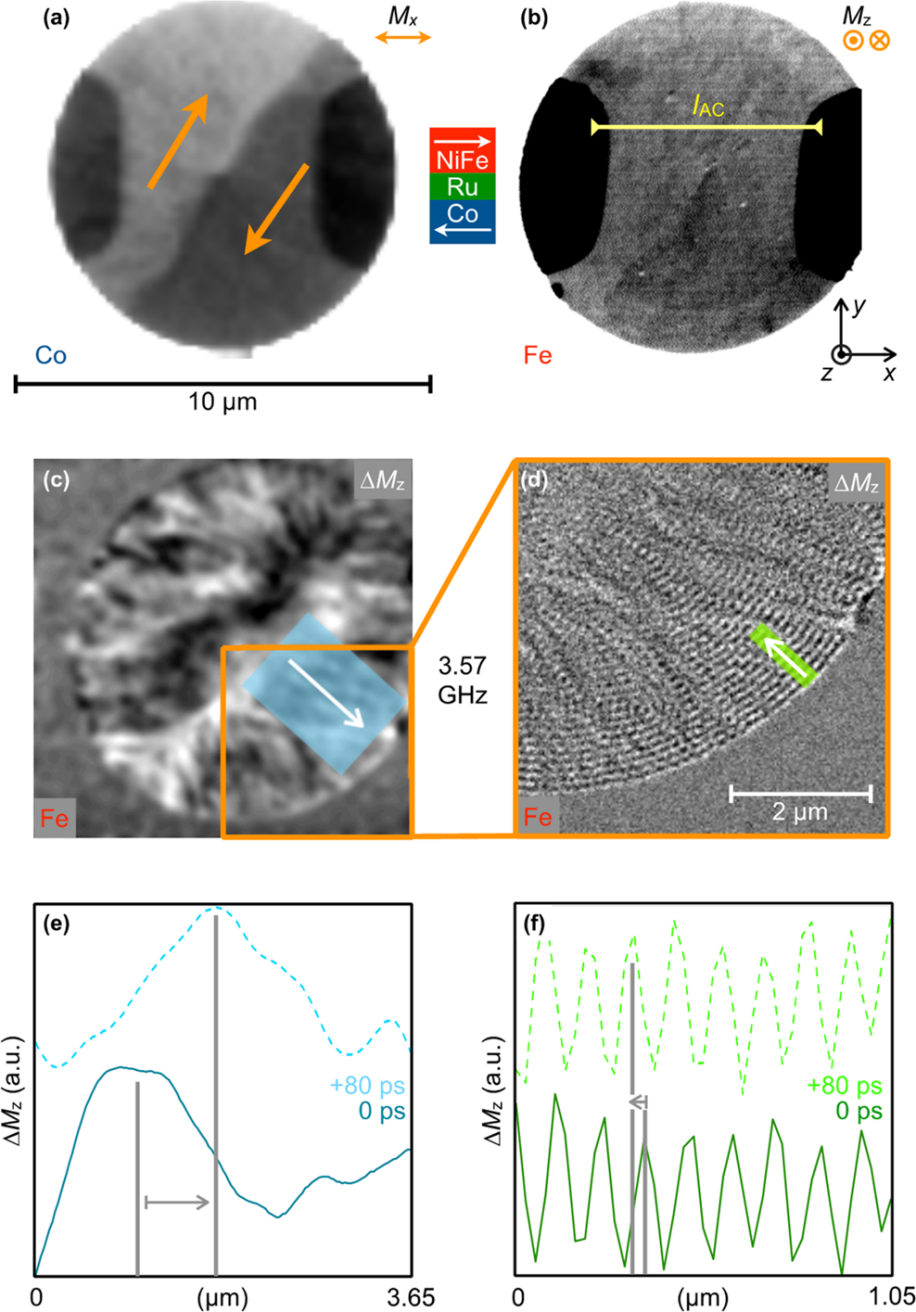
TR-STXM measurement of coherent, nonreciprocal spin waves. (a) STXM image with in-plane magnetic sensitivity (∼*M*_*x*_) at the Co L_3_ edge. (b) STXM image with perpendicular magnetic sensitivity (∼*M*_*z*_) at the Fe L_3_ edge; the domain wall and vortex core are visible. Electric Cu contacts for injecting alternating currents flowing laterally through the structure for dynamic excitation can be seen as dark absorption contrast areas on the left and right sides of the disk in (a) and (b). Normalized response (c) (snapshot in time) of the sample ∼*Δm*_*z*_(Fe) at 3.57 GHz showing outward propagating waves with long wavelengths. (d) Magnified image ∼Δ*M*_*z*_(Fe) at 3.57 GHz showing inward propagating waves with short wavelengths. (e) Averaged line profiles [blue box in (c), along white arrow] of the long waves at a time delay of 80 ps, (f) the same for the short waves averaged over the green box in (d).

The sample investigated was a disk of ∼9 μm diameter, fabricated out of a stack of magnetron sputtered Co(47.8 nm)/Ru(0.8 nm)/Ni_81_Fe_19_(44.9 nm),^32^ using electron beam lithography and ion beam etching on a soft X-ray transparent SiN membrane substrate (see Methods). The nonferromagnetic Ru interlayer mediates an antiferromagnetic interlayer exchange coupling between the two ferromagnetic layers of Co and NiFe.^27^ For excitation purposes, 200 nm thick Cu interconnections were deposited at two opposing sides of the structure by using electron beam lithography, electron beam evaporation, and lift-off. The interconnections were overlapping with the disk by about 2 μm on each side; see Figure 2(a,b).

For identifying the remanent local magnetic state of the sample, we used scanning transmission X-ray microscopy (STXM)^39^ (see Methods), exploiting the X-ray magnetic circular dichroism effect for magnetic contrast.^40^ This method allows for a lateral spatial resolution of ∼25 nm, while it is also layer-selective for the given magnetic system via element-specific resonant X-ray absorption. The measurements were carried out at the Maxymus end station^39,50^ at the BESSYII electron storage ring operated by the Helmholtz-Zentrum Berlin für Materialien and Energie. The magnetic state of the disk was found to be in an anisotropically distorted bidomain vortex state supporting a 180° domain wall [see Figure 2(a)],^33,41^ with all-antiparallel orientation of the in-plane magnetization between the two layers. This domain pattern can be clearly seen in the micrograph with partial in-plane magnetic sensitivity (∼*M*_*x*_) recorded at the Co L_3_ edge [Figure 2(a)] but also has its signature in the image in Figure 2(b), recorded at the Fe L_3_ edge with pure perpendicular magnetic sensitivity (∼*M*_*z*_) [cf. Supporting Information (SI) (1)].

It was shown earlier that vortex cores, as well as domain walls, can be used to excite short-wavelength spin waves when driven nonresonantly by alternating magnetic fields.^32,33,42−44^ Here, we use sinusoidally alternating currents *I*(*t*) = *I* cos(2π*ft*) flowing through the structure itself to drive the excitation of the given magnetic textures via a combination of spin-transfer-torques^45,46^ and internal Oersted fields, the details of which will be discussed separately.^47^ The response of the sample to this excitation was directly imaged in time-resolved (TR)-STXM^48,49^ for various excitation frequencies up to 5 GHz (see Methods). Figure 2, panels c and d show the resulting spin-wave pattern generated by the vortex spin texture for an excitation frequency of ∼3.5 GHz and an excitation current of *I* = 34 mA (corresponding to an approximate current density of 4 × 10^10^A/m^2^). These normalized snapshots display the relative change of the perpendicular magnetization over time Δ*M*_*z*_(*t*)/<*M*_*z*_>_*t*_ recorded at the Fe L_3_ edge. In view of the full sample, long-wavelength spin waves (λ ≈ 3.3 μm) can be observed [Figure 2(c)] to propagate outward with a phase velocity of *v*_ph_ ≈ 10 km/s, as supported by the averaged line profiles shown in Figure 2(e) for two different time steps, delayed by 80 ps, and by the Supporting Movie (M1). Simultaneously, short-wavelength spin waves are propagating inward [see high-resolution micrograph in Figure 2(d)] with λ ≈ 125 nm and a phase velocity of *v*_ph_ ≈ 440 m/s, as it can be seen again by the corresponding average line profiles, Figure 2(f), and the Supporting Movie (M1). This experimental result provides direct evidence for a significant nonreciprocity (λ^–^/λ^+^ > 10|_f=3.5 GHz_) of layer-collective coherent spin waves in a system of antiparallel-oriented magnetic bilayers. Note that the spin waves are mainly excited by the oscillation of the central domain wall to propagate outward through the long-wavelength branch (*k*^–^) [cf. A^–^ in Figure 1(c)]. Upon reflection at the edge of the disk, these waves are then propagating back inward via the short-wavelength branch (*k*^+^) [cf. A^+^ in Figure 1(c)]. In line with that, the propagation sense of both spin-wave branches would be reversed when the in-plane circulation configurations of both vortices were opposite [see SI (1) and Supporting Movie M5]. Note that while alternating currents are used here for the spin-wave excitation, the resulting spin-wave dynamics and nonreciprocity are independent of the specific excitation mechanism (that, e.g., could also be via magnetic fields). In particular, we do not observe any noticeable direct effect from the alternating current on the spin-wave propagation or dispersion relation. There is also no specific influence of the electrodes on the spin-wave dynamics in the regions where the electrodes overlap with the disk sample. While the strongly nonreciprocal waves observed here are part of the acoustic layer-collective spin-wave spectrum of the bilayer system, there may also be—in principle—optical layer-collective waves, yet these are predicted to occur at higher frequencies and with a much lower nonreciprocity^33,16^ [cf. SI (3)].

**Figure 3:**
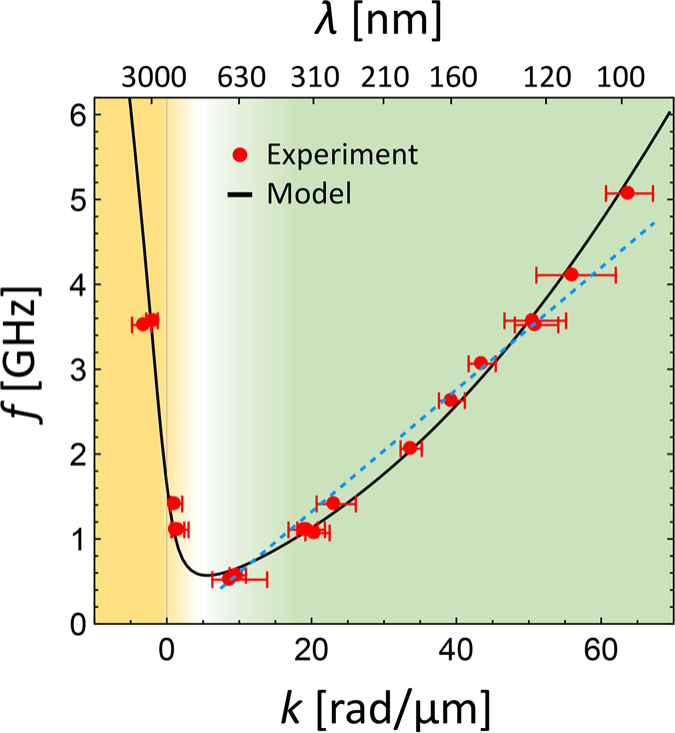
Nonreciprocal magnon dispersion relation *f*(*k*) in an antiparallel magnetic bilayer. Experimental data points (red dots), analytic calculation (black solid line), and linear approximation of slow branch (blue dashed line). Green background indicating slow branch (*k*^+^) and orange background indicating fast branch (*k*^–^).

For a more detailed understanding of the spin-wave nonreciprocity in the system in remanence, we analyzed the corresponding spin-wave dispersion relation *f*(*k*), as it is shown in Figure 3. Red circles correspond to the experimentally determined *k*-values (from measuring λ and the phase propagation direction) at different frequencies. Thereby, a clear nonreciprocal spin-wave dispersion can be identified: for *k* ≥ + 8 rad/μm, there is a short wavelength spin-wave branch (*k*^+^) (green background), whereas a long-wavelength branch ranges from ≲ +2 rad/μm to negative *k*-values (orange background). This experimental evidence shows that the minimum frequency of the dispersion is approximately 500 MHz, corresponding to a *k-*value of assumedly +8 rad/μm, i.e., displaced from zero. While the group velocity is zero at this point, corresponding to nonpropagating spin waves, both phase and group velocity are always positive for the short-wavelength branch, assuming a monotonous dispersion with a single minimum. However, the long-wavelength branch exhibits either positive or negative phase velocities at always negative group velocity. See also the Supporting Movie (M2) with an inward propagating phase yet outward propagating energy flow for 0 < *k* < + 2 rad/μm inferred [cf. A^–^ vs B^–^ in Figure 1(c)]. Overall, the observed spin waves exhibit wavelengths from 5 μm down to 100 nm in a frequency range from 500 MHz to 5 GHz.

In order to gain further insight into the spin-wave dispersion, we applied an analytic model for plane waves in continuous lateral films using the dynamic matrix method,^50,51^ which involves the subdivision of the magnetic medium into smaller cells, in this case, *N* sublayers^33^ (see Methods). Thus, *N* coupled Landau–Lifshitz equations are considered.^52^
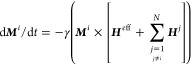
Here, ***M***^*i*^ corresponds to the magnetization of the *i*th discretization layer, ***H***^eff^ is the intralayer effective field, and ***H***^*j*^ is the field from the *j*th discretization layer (*j* ≠ *i*), γ = 2*πgμ*_B_/*h* is the gyromagnetic ratio with *g* being the *g*-factor (considering 2.12 as average value of the two layers: NiFe 2.10, Co 2.14^53^), μ_B_ being the Bohr magneton, and *h* being the Planck constant. The corresponding linearized eigen problem was solved numerically under variation of the following parameters: Exchange constant (*A*), intrinsic uniaxial in-plane anisotropy (*K*_u_) and bilinear interlayer exchange coupling constant (*J*_L_). Magnetic properties leading to a good fit of the experimental data are composed in Table 1, with *d* corresponding to the layer thickness inferred by transmission electron microscopy and *M*_s_ corresponding to the saturation magnetization both not being subject to variation.^54^

**Table 1:** Magnetic Properties of the Co/Ru/NiFe Stack

	*A* (pJ/m)	*M*_s_ (MA/m)	*K*_u_ (J/m^3^)	*d* (nm)	*J*_L_ (mJ/m^2^)
Co	15	1.27	1900	47.8	-
NiFe	9	0.74	750	42.8	-
Ru	-	-	-	0.8	–0.1

Considering these magnetic parameters shown in Table 1, the calculated dispersion relation matches well with the experimental data points (see Figure 3, black line), supporting our interpretation of the experimental findings. Note that the exchange constants considered here are significantly lower than the reference values for single crystalline materials; nevertheless, they compare well with recent reports for polycrystalline thin films.^18,55^ Earlier studies also confirmed that the type of calculations made here agree well with corresponding micromagnetic simulations^33,35^ and that the plane wave assumption holds for waves in finite structures when the wavelength is below or on the order of the effective structure size.^18,33^

By means of the calculated dispersion curve, further conclusions can be drawn on the spin-wave nonreciprocity in the system investigated: The specific displacement of the dispersion minimum from *k* = 0 is *k* = +5 rad/μm at a frequency gap of 500 MHz, and this displacement is a result of both anisotropy and layer asymmetry (Co, NiFe). Besides the already quantified λ(*f*_0_) wavelength nonreciprocity for a particular frequency, also the more general nonreciprocity in group velocity can be determined to be of the same very high order of ≈10 [|*v̅*_g^–^_|/*v̅*_g^+^_ ≈ (4400 m/s)/(460 m/s)], when linearly approximating the calculated dispersion curves (blue dashed line for the *k*^+^ branch in Figure 3). Note that apart from the direct vicinity of the minimum, the two dispersion branches are to some extent linear yet they exhibit a slight parabolic contribution for higher *k*-values in the short-wavelength branch (as a result of an increasing influence of exchange interaction). More general, the following qualitative trends can be determined from the calculations of our antiparallel magnetic bilayer system: The magnon nonreciprocity, induced by the dynamic dipolar interaction, increases with the thickness of the ferromagnetic layers, while it decreases with the thickness of the interlayer, and both the fundamental frequency gap and the *k*-shift of the minimum increase with magnetic anisotropy. These trends substantiate our rationale of choosing a stack with relatively thick ferromagnetic layers and a thin nonferromagnetic interlayer that ensures antiparallel orientation. Note that the set of magnetic parameters used here represents one particular solution of the problem, while other sets with slightly different parameters may also lead to good fits of the experimental data. In that respect, the relatively high value of *K*_u_ for NiFe is noteworthy, which on the other hand might also be a consequence of the microfabrication processing, in particular of the ion beam etching or oxygen plasma steps. In order to provide an understanding of the effects of individual parameters on the spin-wave dispersion relation, we have included the results from calculations with varying parameters in SI (4). From these calculations, it can be concluded that the general properties of the dispersion relation do not critically depend on individual parameters or their combination but are rather universal characteristics of the antiparallel bilayer system. In general terms, our antiparallel magnetic bilayer system can be categorized as a biased, linear, time-invariant nonreciprocal media, where time-inversion symmetry breaking stems from the (self)-bias of the magnetic orientation of the individual magnetic layers.^1^

**Figure 4:**
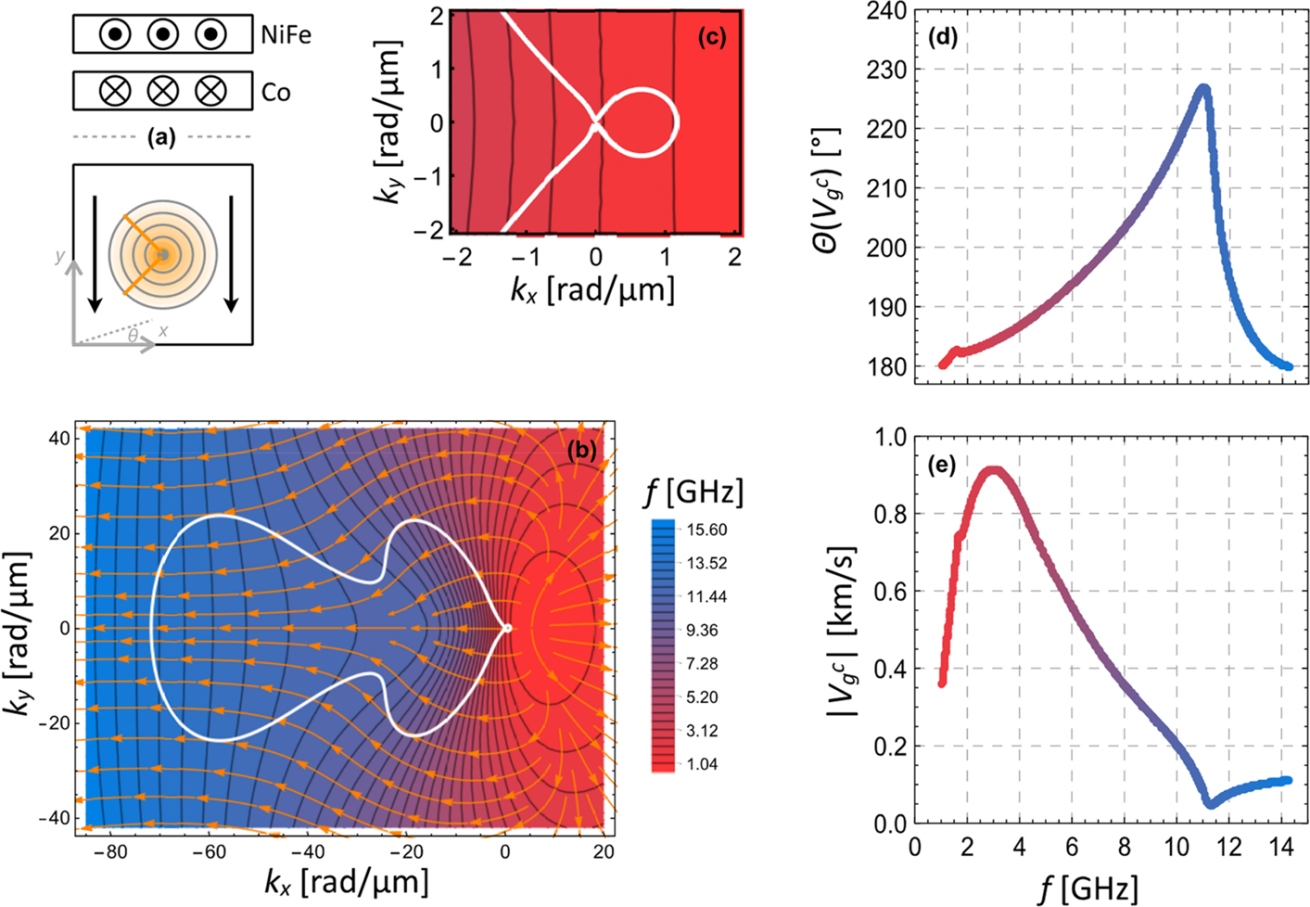
Caustic spin-wave self-focusing in an antiparallel magnetic bilayer. (a) Schematics: top: cross-section of the static magnetic orientation in the lower Co and upper NiFe layer; bottom: spin-wave emission from a point-source with orange caustic beams and coordinate system (top view). (b) Calculated frequency map (color scale at the right) of the lowest energy magnon band with isofrequency lines for lateral wavevectors ***k***_*xy*_. The local group velocity direction is indicated by orange arrows, and caustic beams occur along the white solid contour. (c) Zoom-in of small subcaustic around *k* = (0,0). (d,e) Group velocity ***v***_g_^c^ of caustic beams as a function of frequency: (d) Angle between ***v***_g_^c^ and −**e**_*x*_, (e) absolute value |***v***_g_^c^|.

Given such a system with strong spin-wave nonreciprocity, we want to outline a distinct scenario for its utilization in magnonic applications, namely the caustic self-focusing of spin waves. We choose this particular scenario as the occurrence of caustic beams appeared to be highly promising from the underlying dispersion relation.^56^ Here, caustic focusing means that the power flow of waves originating from a point source is not isotropic in space but rather confined to certain directions (beams) [cf. Figure 4(a)] as a consequence of an anisotropic dispersion relation of the hosting medium. A necessary condition for such a caustic effect is that, in vectorial notation, ***v***_ph_ and ***v***_g_ are not parallel for certain ***k***. More specifically, caustic beams occur at any fixed frequency (*f*_0_) and wavevector ***k*** for which the corresponding isofreqency surface ***k***(*f*_0_) (also termed slowness surface) has zero curvature. While caustic self-focusing was first observed for phonon transport in solid crystals,^57,58^ it was subsequently transferred to magnon transport in thin films enabling the controlled excitation of confined spin-wave beams in two dimensions ***k*** = (*k*_*x*_,*k*_*y*_).^56,59−64^

To analyze potential caustic effects in the antiparallel magnetic bilayer system, we numerically calculated the two-dimensional isofrequency contours ***k***(*f*_0_) of the first magnon band in the range [(−85 ≤ *k*_*x*_ ≤ 20),(−45 ≤ *k*_*y*_ ≤ 45)] rad/μm from the analytic theory; see Figure 4(b) (color scale). Note that by the first magnon band we refer to the lowest energy, continuous spin-wave dispersion hyperplane (*f*,*k*_*x*_,*k*_*y*_), potentially changing its mode character at avoided crossings with higher-order modes for frequencies above the experimentally addressed range. See SI (5) for further details about the dispersion of higher-order spin-wave modes. Specifically, the first spin-wave band is the hyperplane spanned between the black curves of Figure S4a,b. Within this isofrequency plot of Figure 4(a), orange arrows indicate the orientation of the calculated local spin-wave group velocity, ***v***_g_(*k*_*x*_,*k*_*y*_) as a measure for the direction of power flow, which is always perpendicular to the isofrequency contour. By analyzing the curvature of the isofrequency contours using the formalism described in SI (6), we can identify caustic points of zero curvature in a frequency range between approximately 1 and 14.5 GHz. These points form closed caustic contours symmetric to the *k*_*x*_-axis, as indicated by the white curve in Figure 4(b). The main caustic contour extends from about (0,0) to *k*_*x*_ ∼ −70 rad/μm, covering *k*_*y*_ values symmetrically up to (±25) rad/μm. This caustic contour exhibits a *k*_*y*_-constriction of ∼10 rad/μm, asymmetrically over (−60 ≤ *k*_*x*_ ≤ −20) rad/μm, which leads to an overall shape similar to a “porcino”. In addition to that, we see that close to the *k*-origin there is another small circular caustic feature arising in the contour, see Figure 4(c), in the range of (*k*_*x*_: 0...1|*k*_*y*_: −0.5...0.5) rad/μm.

Each point (*f*,*k*_*x*_,*k*_*y*_)^c^ on the caustic contour (*c*) corresponds to a spin-wave beam with a specific local caustic group velocity ***v***_g_^c^. In Figure 4(d) the angle θ of ***v***_g_^c^ relative to **e**_*x*_ is plotted as a function of frequency, for the ***v***_g_^c^-branch in upper *k*_*y*_ half-space. This angle is 180° at its lowest and highest frequencies (1.0 and 14.5 GHz), corresponding to a negative group velocity (along −**e**_*x*_). Note that apart from these frequencies, where there is a single beam only, two simultaneous caustic beams exist symmetrically to the *x*-axis for all intermediate frequencies. Apart from a small nonmonotonicity around 1.5 GHz (the extent of which may sensitively depend on the specific magnetic parameters), θ increases up to around 225° at 11 GHz before it more sharply decreases back to 180° at 14.5 GHz. Correspondingly, Figure 4(e) displays the absolute value |***v***_g_^c^| as a function of frequency, revealing alternating behavior. Starting from 0.35 km/s at 1 GHz, it first increases to 0.9 km/s at 3 GHz, decreases back to 0.05 km/s at 11 GHz, and, finally, reaches 0.1 km/s at 14.5 GHz. The results from the calculations above indicate that the system of antiparallel magnetic bilayers could indeed be highly suitable to exploit self-focusing caustic effects, as the predicted beams occur over a much wider range of frequencies and wavenumbers compared to single-layer films^56^ and they appear to be highly tunable. A detailed analysis of the role of thickness and magnetic graduation on the caustic properties of bilayers is reported elsewhere.^65^

## Conclusions

We have directly observed coherent spin-wave nonreciprocity in a (Co/Ru/NiFe) functional magnetic bilayer with antiparallel magnetizations and magnetic anisotropy using TR-STXM imaging. The corresponding spin waves were imaged in a system of two stacked distorted vortices (diameter ∼9 μm) with opposite in-plane vorticities, at frequencies between 500 MHz and 5 GHz and with wavelengths from 5 μm down to 100 nm. For waves of the layer-collective eigenmode in the Damon–Eshbach geometry, we found a strong nonreciprocity with a slowly propagating short-wavelength branch versus a fast and counter-propagating long-wavelength branch. Apart from the region of the dispersion minimum, both wavelengths and linearized group velocities exhibit a high nonreciprocity factor of about 10 or above within the experimentally addressed range (*f*,*k*). A *k*-shift of the dispersion minimum causes the fast spin-wave branch to have either positive (*k* > 0) or negative phase velocities (*k* < 0) at almost always negative group velocities, while for the slow spin-wave branch both phase and group velocities are positive at all times. Theoretical calculations of the spin-wave dispersion support our experimental findings. Moreover, these calculations predict the occurrence of broadband caustic spin-wave focusing effects in antiparallel magnetic bilayers. For the lowest magnon band, self-focusing spin-wave beams arise at frequencies in the range of 1 to 14.5 GHz with wavenumbers up to 70 rad/μm, and their predicted emission angles and group velocities are strongly frequency dependent. In perspective, while the nonreciprocity factor found in this work is much higher than the ones observed earlier, it may still be increased by tailoring the design of the layer stack (for example, by reducing the interlayer thickness) and by exploiting curvilinear effects.^66,67^ Such spin-wave nonreciprocities could be exploited for the realization of spin-wave isolator or circulator devices. Note, however, that the purpose of the present study was to demonstrate nonreciprocal coherent spin-wave propagation on a basic scientific level and that for real-world devices likely other ways of implementation are required in terms of functional areal density and spin-wave excitation efficiency. Therefore, the results of our study are mainly of fundamental relevance, yet they may also stimulate the development of magnonic applications based on spin-wave nonreciprocity and caustic effects.

## Methods

### Sample Fabrication

The magnetic multilayer was deposited onto an X-ray transparent silicon-nitride window membrane (200 nm thickness, 500 × 500 μm^2^ window area) by the use of magnetron sputtering. The magnetic stack was capped by an aluminum layer of 3 nm thickness for oxidation protection, The micrometer-sized disk was patterned by means of electron beam lithography (EBL) and consecutive ion beam etching. To this end, at first a negative resist (MA-N 2910) was spun onto the film upon an initial oxygen plasma treatment for optimizing adhesion and baked out. Second, the disk area was exposed by EBL, and the samples were developed for 300 s in MA-D 525 and subsequently rinsed in deionized water (60 s). During the final step, the sample was milled using a wide argon ion beam at two different angles (85° and 5°) for approximately 1 h to physically etch the disk out of the continuous film. For removing the remaining resist, an acetone bath (12 h) and a second oxygen plasma step (20 min) were applied. For electric contacting, two copper/aluminum leads of 200/5 nm thickness were additionally fabricated, each overlapping with the disk for about 2 μm at opposing rim positions (see Figure 2). The leads were patterned using positive resist EBL, electron beam evaporation, and lift-off processing.

### STXM

Synchrotron-based scanning transmission X-ray microscopy (STXM) was used to image the magnetic orientation in the multilayer disk. For that purpose, a Fresnel zone plate was employed to focus monochromatic X-rays onto the sample, while undiffracted X-rays and those of higher diffraction order are blocked by the central stop of the zone plate and a circular order selecting aperture. The X-ray intensity transmitted through the sample is measured by a single pixel detector. Raster scanning of the sample through the focused beam generates an image with approximately 25 nm lateral resolution. Magnetic contrast is provided by using circularly polarized X-rays via the X-ray magnetic circular dichroism (XMCD).^40^ Tuning the incident photon energy to a specific resonant absorption edge of a particular chemical element allows for selective probing of the magnetic orientation of this element. For the given magnetic bilayer, therefore, the two layers can be separately imaged in terms of magnetic orientation by measuring at photon energies of Co L_3_ ≈ 778 eV and Fe L_3_ ≈ 708 eV or Ni L_3_ ≈ 853 eV, respectively. The logarithmic magnetic transmission contrast detected is proportional to the projection of the magnetization on the photon propagation direction, which means that in normal incidence STXM is sensitive to the perpendicular magnetization component, while inclination of the sample allows for accessing in-plane magnetization components as well.

### TR-STXM

The magnetization dynamics of the multilayer disks was imaged by means of stroboscopic time-resolved STXM (TR-STXM).^39^ For that purpose, the time structure of the incident X-ray pulses is exploited at a 500 MHz repetition rate and ∼100 ps effective pulse width, leading to a frequency resolution of approximately 5 GHz. The accessible frequencies are of the form *f* = *Q* * 500 MHz/*S*, where *Q* is an integer multiplier and *S* corresponds to the integer number of phases simultaneously acquired for a sinusoidal excitation. The excitation signal was measured both in front of (via a −20 dB pick-off tee) and behind the sample by an oscilloscope. The excitation current densities at the sample were on the order of 10^9^ to 10^10^ A/m^2^.

Spin-wave wavelengths at different frequencies were determined from the TR-STXM images by analyzing lateral line profiles. With respect to the imaging uncertainty of TR-STXM, the frequency error is below 10 MHz, while errors in *k* originate from the wavelength uncertainties as the measurement technique applies to real space. For wavelengths below 1 μm, we accounted for an error of 3 image pixels divided by the number of waves considered. For that reason, higher *k* values, by trend, have larger errors, and the error of a particular *k* is asymmetric with larger errors toward the direction of higher *k*. For wavelengths below half of the diameter of the structure (1 μm < λ < 4 μm), we considered an error of (+50%, −33%) in the estimation, while for wavelengths above 4 μm, a possible upper uncertainty boundary of (+100%, −50%) was taken into account.

### Micromagnetic Calculations

The dynamic matrix method is employed to calculate the spin-wave dynamics.^50,51^ In this approach, the synthetic ferrimagnetic system is divided into many sublayers to account for the thickness dependence of the spin-wave modes. The temporal evolution of the magnetization is given by the Landau–Lifshitz (LL) equation of motion,^52^ namely, **M**^(ν)^ = −μ_0_γ**M**^(ν)^ × **H**^*e*(ν)^. Here, **M**^(ν)^ and **H**^*e*(ν)^ denote the magnetization and effective field of sublayer ν, respectively, and γ is the magnitude of the gyromagnetic ratio. A local coordinate system (*X*_ν_, *Y*_ν_, *Z*_ν_) is used in the calculations, where the *X*_ν_ axis aligns with the equilibrium magnetization of the sublayer ν, the *Z*_ν_ axis is perpendicular to the films, and the *Y*_ν_ axis lies within the plane of the films. Note that *X*_ν_ allows the definition of the equilibrium magnetization of each sublayer such that it is possible to consider an antiparallel alignment of the magnetizations, which characterizes the synthetic ferrimagnet structure. For small oscillations of the magnetization around the equilibrium state, the equation of motion can be expressed as follows,
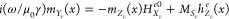
1and
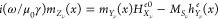
2where *M*_*S*_ν__ is the saturation magnetization of the νth layer and *H*_*X*_ν__^*e*0^ is the *X*_ν_-component of the equilibrium effective field. Besides, **m**(*x*) = *m*_*Y*_ν__ (*x*)*Ŷ*_ν_ + *m*_*Z*_ν__ (*x*)*Ẑ*_ν_ represents the dynamic magnetization, where it has been assumed that **m**(*x*) = **m**(*x*)*e*^*iωt*^ with ω = 2*πf* denoting the angular frequency. The dependence of the dynamic magnetization on the *x*-coordinate is attributed to the assumption that spin-wave propagation occurs along the *x* axis; this is **m**(*x*) = **m**_**k**_(*x*)*e*^*ikx*^, where *k* represents the wave vector. Finally, eqs 1 and 2 can be formulated as an eigenvalue problem, which can be expressed as,
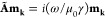
3

The matrix elements of **Ã** are associated with the energetic interactions within the system. In the present scenario, these interactions are Zeeman, demagnetizing, in-plane uniaxial anisotropy, intralayer exchange, and interlayer terms that interconnect the magnetic sublayers. Detailed information about these interactions and their corresponding matrix elements can be found in Appendix A.3 of ref (68).

The exchange interaction between sublayers is considered through the associated energy density given by
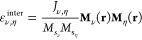
4

In the case that the sublayers ν and η correspond to the ones located at the interface that separates the ferromagnetic layers, the interlayer exchange constant becomes *J*_ν,η_ = *J*_L_, being *J*_L_ < 0 to stabilize the antiparallel alignment of the magnetizations. For all other cases, it can be shown that *J*_ν,ν+1_ = 2*A*_ν_/*d*, with *A*_ν_ being the exchange constant of the νth sublayer and *d* its thickness.

## Supporting Information Available

(1) Static domain configurations and antiferromagnetic interlayer coupling (with Figure S1). (2) Supporting TR-STXM movies. (3) Acoustic and optic spin-wave modes (with Figure S2). (4) Effect of the magnetic parameters on the spin-wave dispersion relation (with Figure S3). (5) Higher order spin wave modes and bands (with Figure S4). (6) Identifying points of zero curvature and the lines these points form in the isofrequency contours of the spin-wave dispersion relation *f(k*_*x*_,*k*_*y*_) (with 5 mathematical formulas) (PDF)
Five Supporting Movies (M1-M5) (AVI-2, AVI-2, AVI-3, AVI-4, AVI-5)

## Supplementary Material

nn3c08390_si_001.pdf

nn3c08390_si_002.avi

nn3c08390_si_003.avi

nn3c08390_si_004.avi

nn3c08390_si_005.avi

nn3c08390_si_006.avi

## References

[ref1] Caloz C.; Alu A.; Tretyakov S.; Sounas D.; Achouri K.; Deck-Leger Z.-L. Electromagnetic Nonreciprocity. Physical Review Applied 2018, 10 (4), 047001 10.1103/PhysRevApplied.10.047001.

[ref2] Sounas D. L.; Caloz C.; Alu A. Giant non-reciprocity at the subwavelength scale using angular momentum-biased metamaterials. Nat. Commun. 2013, 4, 2407 10.1038/ncomms3407. 23994940

[ref3] Reiskarimian N.; Krishnaswamy H. Magnetic-free non-reciprocity based on staggered commutation. Nat. Commun. 2016, 7, 11217 10.1038/ncomms11217. 27079524 PMC4835534

[ref4] Yu Z. F.; Fan S. H. Complete optical isolation created by indirect interband photonic transitions. Nat. Photonics 2009, 3 (2), 91–94. 10.1038/nphoton.2008.273.

[ref5] Ruter C. E.; et al. Observation of parity-time symmetry in optics. Nat. Phys. 2010, 6 (3), 192–195. 10.1038/nphys1515.

[ref6] Sounas D. L.; Alu A. Non-reciprocal photonics based on time modulation. Nat. Photonics 2017, 11 (12), 774–783. 10.1038/s41566-017-0051-x.

[ref7] Fang K. J.; et al. Generalized non-reciprocity in an optomechanical circuit via synthetic magnetism and reservoir engineering. Nat. Phys. 2017, 13 (5), 465–471. 10.1038/nphys4009.

[ref8] Fleury R.; et al. Sound Isolation and Giant Linear Nonreciprocity in a Compact Acoustic Circulator. Science 2014, 343 (6170), 516–519. 10.1126/science.1246957. 24482477

[ref9] Bloch F. Zur Theorie des Ferromagnetismus. Zeitschrift Fur Physik 1930, 61 (3–4), 206–219. 10.1007/BF01339661.

[ref10] Kruglyak V. V.; Demokritov S.O.; Grundler D. Magnonics. J. Phys. D: Appl. Phys. 2010, 43 (26), 264001 10.1088/0022-3727/43/26/264001.

[ref11] Chumak A. V.; et al. Magnon spintronics. Nat. Phys. 2015, 11 (6), 453–461. 10.1038/nphys3347.

[ref12] Chumak A. V.; et al. Roadmap on Spin-Wave Computing. IEEE Trans. Magn. 2022, 58 (6), 1 10.1109/TMAG.2022.3149664.

[ref13] Barman A.; Gubbiotti G.; Ladak S; Adeyeye A O; Krawczyk M; Grafe J; Adelmann C; Cotofana S; Naeemi A; Vasyuchka V I; Hillebrands B; Nikitov S A; Yu H; Grundler D; Sadovnikov A V; Grachev A A; Sheshukova S E; Duquesne J-Y; Marangolo M; Csaba G; Porod W; Demidov V E; Urazhdin S; Demokritov S O; Albisetti E; Petti D; Bertacco R; Schultheiss H; Kruglyak V V; Poimanov V D; Sahoo S; Sinha J; Yang H; Munzenberg M; Moriyama T; Mizukami S; Landeros P; Gallardo R A; Carlotti G; Kim J-V; Stamps R L; Camley R E; Rana B; Otani Y; Yu W; Yu T; Bauer G E W; Back C; Uhrig G S; Dobrovolskiy O V; Budinska B; Qin H; van Dijken S; Chumak A V; Khitun A; Nikonov D E; Young I A; Zingsem B W; Winklhofer M The 2021 Magnonics Roadmap. J. Phys.: Condens. Matter 2021, 33 (41), 413001 10.1088/1361-648X/abec1a. 33662946

[ref14] Gurevich A. G.; Melkov G. A. Magnetization Oscillations and Waves; CRC Press: Boca Raton, 1996.

[ref15] Camley R. E. Nonreciprocal Surface-Waves. Surf. Sci. Rep. 1987, 7 (3–4), 103–187. 10.1016/0167-5729(87)90006-9.

[ref16] Damon R. W.; Eshbach J. R. Magnetostatic Modes of a Ferromagnet Slab. J. Phys. Chem. Solids 1961, 19 (3–4), 308–320. 10.1016/0022-3697(61)90041-5.

[ref17] Kostylev M. Non-reciprocity of dipole-exchange spin waves in thin ferromagnetic films. J. Appl. Phys. 2013, 113 (5), 053907 10.1063/1.4789962.

[ref18] Dieterle G.; et al. Coherent Excitation of Heterosymmetric Spin Waves with Ultrashort Wavelengths. Phys. Rev. Lett. 2019, 122 (11), 117202 10.1103/PhysRevLett.122.117202. 30951356

[ref19] Zakeri K.; Zhang Y.; Prokop J.; Chuang T.-H.; Sakr N.; Tang W. X.; Kirschner J., Asymmetric Spin-Wave Dispersion on Fe(110): Direct Evidence of the Dzyaloshinskii-Moriya Interaction. Phys. Rev. Lett. 2010, 104, (13), . 10.1103/PhysRevLett.104.137203 20481909

[ref20] Di K.; Zhang V. L.; Lim H. S.; Ng S. C.; Kuok M. H.; Yu J.; Yoon J.; Qiu X.; Yang H. Direct Observation of the Dzyaloshinskii-Moriya Interaction in a Pt/Co/Ni Film. Phys. Rev. Lett. 2015, 114 (4), 047201 10.1103/PhysRevLett.114.047201. 25679905

[ref21] Dzyaloshinsky I. A Thermodynamic Theory of Weak Ferromagnetism of Antiferromagnetics. J. Phys. Chem. Solids 1958, 4 (4), 241–255. 10.1016/0022-3697(58)90076-3.

[ref22] Moriya T. Anisotropic Superexchange Interaction and Weak Ferromagnetism. Phys. Rev. 1960, 120 (1), 91–98. 10.1103/PhysRev.120.91.

[ref23] Zingsem B. W.; Farle M.; Stamps R. L.; Camley R. E. Unusual nature of confined modes in a chiral system: Directional transport in standing waves. Phys. Rev. B 2019, 99 (21), 047201 10.1103/PhysRevB.99.214429.

[ref24] Schneider T.; Serga A. A.; Neumann T.; Hillebrands B.; Kostylev M. P. Phase reciprocity of spin-wave excitation by a microstrip antenna. Phys. Rev. B 2008, 77 (21), 214411 10.1103/PhysRevB.77.214411.

[ref25] Grunberg P. Magnetostatic Spin-Wave Modes of a Heterogeneous Ferromagnetic Double-Layer. J. Appl. Phys. 1981, 52 (11), 6824–6829. 10.1063/1.328636.

[ref26] Mika K.; Grunberg P. Dipolar Spin-Wave Modes of a Ferromagnetic Multilayer with Alternating Directions of Magnetization. Phys. Rev. B 1985, 31 (7), 4465–4471. 10.1103/PhysRevB.31.4465. 9936378

[ref27] Grunberg P.; et al. Layered Magnetic-Structures - Evidence for Antiferromagnetic Coupling of Fe Layers across Cr Interlayers. Phys. Rev. Lett. 1986, 57 (19), 2442–2445. 10.1103/PhysRevLett.57.2442. 10033726

[ref28] Nortemann F. C.; Stamps R. L.; Camley R. E. Microscopic Calculation of Spin-Waves in Antiferromagnetically Coupled Multilayers - Nonreciprocity and Finite-Size Effects. Phys. Rev. B 1993, 47 (18), 11910–11923. 10.1103/PhysRevB.47.11910. 10005363

[ref29] Stamps R. L. Spin Configurations and Spin-Wave Excitations in Exchange-Coupled Bilayers. Phys. Rev. B 1994, 49 (1), 339–347. 10.1103/PhysRevB.49.339. 10009291

[ref30] Kabos P.; et al. Brillouin Light-Scattering on Fe/Cr/Fe Thin-Film Sandwiches. J. Appl. Phys. 1994, 75 (7), 3553–3563. 10.1063/1.356092.

[ref31] Di K.; Feng S. X.; Piramanayagam S. N.; Zhang V. L.; Lim H. S.; Ng S. C.; Kuok M. H., , Enhancement of spin-wave nonreciprocity in magnonic crystals via synthetic antiferromagnetic coupling. Sci. Rep. 2015, 5. 10.1038/srep10153 PMC4423564 25950082

[ref32] Wintz S.; et al. Magnetic vortex cores as tunable spin-wave emitters. Nat. Nanotechnol. 2016, 11 (11), 948–953. 10.1038/nnano.2016.117. 27428277

[ref33] Sluka V.; Schneider T.; Gallardo R. A.; Kakay A.; Weigand M.; Warnatz T.; Mattheis R.; Roldan-Molina A.; Landeros P.; Tiberkevich V.; Slavin A.; Schutz G.; Erbe A.; Deac A.; Lindner J.; Raabe J.; Fassbender J.; Wintz S. Emission and propagation of 1D and 2D spin waves with nanoscale wavelengths in anisotropic spin textures. Nat. Nanotechnol. 2019, 14 (4), 328 10.1038/s41565-019-0383-4. 30804478

[ref34] Mruczkiewicz M.; Graczyk P.; Lupo P.; Adeyeye A.; Gubbiotti G.; Krawczyk M. Spin-wave nonreciprocity and magnonic band structure in a thin permalloy film induced by dynamical coupling with an array of Ni stripes. Phys. Rev. B 2017, 96 (10), 104411 10.1103/PhysRevB.96.104411.

[ref35] Gallardo R.A.; Schneider T.; Chaurasiya A.K.; Oelschlagel A.; Arekapudi S.S.P.K.; Roldan-Molina A.; Hubner R.; Lenz K.; Barman A.; Fassbender J.; Lindner J.; Hellwig O.; Landeros P. Reconfigurable Spin-Wave Nonreciprocity Induced by Dipolar Interaction in a Coupled Ferromagnetic Bilayer. Physical Review Applied 2019, 12 (3), 034012 10.1103/PhysRevApplied.12.034012.

[ref36] Albisetti E.; Tacchi S.; Silvani R.; Scaramuzzi G.; Finizio S.; Wintz S.; Rinaldi C.; Cantoni M.; Raabe J.; Carlotti G.; Bertacco R.; Riedo E.; Petti D. Optically Inspired Nanomagnonics with Nonreciprocal Spin Waves in Synthetic Antiferromagnets. Adv. Mater. 2020, 32 (9), 1906439 10.1002/adma.201906439. 31944413

[ref37] Ishibashi M.; Shiota Y.; Li T.; Funada S.; Moriyama T.; Ono T. Switchable giant nonreciprocal frequency shift of propagating spin waves in synthetic antiferromagnets. Sci. Adv. 2020, 6( (17), ). 10.1126/sciadv.aaz6931 PMC7182415 32494648

[ref38] Jamali M.; Kwon J. H.; Seo S.-M.; Lee K.-J.; Yang H. Spin wave nonreciprocity for logic device applications. Sci. Rep. 2013, 3, 3160 10.1038/srep03160. 24196318 PMC3819604

[ref39] Weigand M.; Wintz S.; Grafe J.; Noske M.; Stoll H.; Van Waeyenberge B.; Schutz G. TimeMaxyne: A Shot-Noise Limited, Time-Resolved Pump-and-Probe Acquisition System Capable of 50 GHz Frequencies for Synchrotron-Based X-ray Microscopy. Crystals 2022, 12 (8), 1029 10.3390/cryst12081029.

[ref40] Schütz G.; et al. Absorption of Circularly Polarized X-Rays in Iron. Phys. Rev. Lett. 1987, 58 (7), 737–740. 10.1103/PhysRevLett.58.737. 10035022

[ref41] Shinjo T.; et al. Magnetic vortex core observation in circular dots of permalloy. Science 2000, 289 (5481), 930–932. 10.1126/science.289.5481.930. 10937991

[ref42] Hermsdoerfer S. J.; Schultheiss H.; Rausch C.; Schafer S.; Leven B.; Kim S.-K.; Hillebrands B. A spin-wave frequency doubler by domain wall oscillation. Appl. Phys. Lett. 2009, 94 (22), 223510 10.1063/1.3143225.

[ref43] Van de Wiele B.; Hamalainen S. J.; Balaz P.; Montoncello F.; van Dijken S. Tunable short-wavelength spin wave excitation from pinned magnetic domain walls. Sci. Rep. 2016, 6, 21330 10.1038/srep21330. 26883893 PMC4756291

[ref44] Hollander R. B.; Muller C.; Schmalz J.; Gerken M.; McCord J. Magnetic domain walls as broadband spin wave and elastic magnetisation wave emitters. Sci. Rep. 2018, 8, 13871 10.1038/s41598-018-31689-8. 30224792 PMC6141534

[ref45] Berger L. Emission of spin waves by a magnetic multilayer traversed by a current. Phys. Rev. B 1996, 54 (13), 9353–9358. 10.1103/PhysRevB.54.9353. 9984672

[ref46] Slonczewski J. C. Current-driven excitation of magnetic multilayers. J. Magn. Magn. Mater. 1996, 159 (1–2), L1–L7. 10.1016/0304-8853(96)00062-5.

[ref47] Koraltan S., , Steerable current-driven emission of spin waves in magnetic vortex pairs. (unpublished). 10.1126/sciadv.ado8635 PMC11423888 39321298

[ref48] Van Waeyenberge B.; et al. Magnetic vortex core reversal by excitation with short bursts of an alternating field. Nature 2006, 444 (7118), 461–464. 10.1038/nature05240. 17122851

[ref49] Acremann Y.; Chembrolu V.; Strachan J. P.; Tyliszczak T.; Stohr J. Software defined photon counting system for time resolved x-ray experiments. Rev. Sci. Instrum. 2007, 78 (1), 014702 10.1063/1.2428274. 17503937

[ref50] Giovannini L.; Montoncello F.; Nizzoli F.; Gubbiotti G.; Carlotti G.; Okuno T.; Shinjo T.; Grimsditch M. Spin excitations of nanometric cylindrical dots in vortex and saturated magnetic states. Phys. Rev. B 2004, 70 (17), 172404 10.1103/PhysRevB.70.172404.

[ref51] Henry Y.; Gladii O.; Bailleul M. Propagating spin-wave normal modes: A dynamic matrix approach using plane-wave demagnetizating tensors. arXiv 2016 [cited 1611.06153; Available from https://arxiv.org/abs/1611.06153 (accessed January 18, 2024).

[ref52] Landau L.; Lifshits E. On the theory of the dispersion of magnetic permeability in ferromgnetic bodies. Phys. Zeitsch. der Sow. 1935. 8.

[ref53] Schoen M. A. W.; Lucassen J.; Nembach H. T.; Koopmans B.; Silva T. J.; Back C. H.; Shaw J. M. Magnetic properties of ultrathin 3d transition-metal binary alloys. I. Spin and orbital moments, anisotropy, and confirmation of Slater-Pauling behavior. Phys. Rev. B 2017, 95 (13), 134411 10.1103/PhysRevB.95.134411.

[ref54] Wintz S.; Strache T.; Korner M.; Bunce C.; Banholzer A.; Monch I.; Mattheis R.; Raabe J.; Quitmann C.; McCord J.; Erbe A.; Lenz K.; Fassbender J. Control of vortex pair states by post-deposition interlayer exchange coupling modification. Phys. Rev. B 2012, 85 (13), 134417 10.1103/PhysRevB.85.134417.

[ref55] Girt E.; Huttema W.; Mryasov O. N.; Montoya E.; Kardasz B.; Eyrich C.; Heinrich B.; Dobin A. Y..; Karis O. A method for measuring exchange stiffness in ferromagnetic films. J. Appl. Phys. 2011, 109 (7), 07B765 10.1063/1.3565203.

[ref56] Kim J. V.; Stamps R.L.; Camley R.E. Spin Wave Power Flow and Caustics in Ultrathin Ferromagnets with the Dzyaloshinskii-Moriya Interaction. Phys. Rev. Lett. 2016, 117 (19), 197204 10.1103/PhysRevLett.117.197204. 27858433

[ref57] Buchwald V. T. Elastic Waves in Anisotropic Media. Proceedings of the Royal Society of London Series a-Mathematical and Physical Sciences 1959, 253 (1275), 563–580.

[ref58] Taylor B.; Maris H. J.; Elbaum C. Phonon Focusing in Solids. Phys. Rev. Lett. 1969, 23 (8), 416 10.1103/PhysRevLett.23.416.

[ref59] Buttner O.; et al. Linear and nonlinear diffraction of dipolar spin waves in yttrium iron garnet films observed by space- and time-resolved Brillouin light scattering. Phys. Rev. B 2000, 61 (17), 11576–11587. 10.1103/PhysRevB.61.11576.

[ref60] Valyavsky A. B.; et al. *Surface Magnetostatic Wave Limited Beam in the Ferrite-Dielectric-Metal Structure.* Radiotekhnika I. Elektronika 1988, 33 (9), 1820–1830.

[ref61] Veerakumar V.; Camley R.E. Magnon focusing in thin ferromagnetic films. Phys. Rev. B 2006, 74 (21), 214401 10.1103/PhysRevB.74.214401.

[ref62] Demidov V. E.; Demokritov S. O.; Birt D.; O’Gorman B.; Tsoi M.; Li X. Radiation of spin waves from the open end of a microscopic magnetic-film waveguide. Phys. Rev. B 2009, 80 (1), 014429 10.1103/PhysRevB.80.014429.

[ref63] Schneider T.; Serga A. A.; Chumak A. V.; Sandweg C. W.; Trudel S.; Wolff S.; Kostylev M. P.; Tiberkevich V. S.; Slavin A. N.; Hillebrands B. Nondiffractive Subwavelength Wave Beams in a Medium with Externally Controlled Anisotropy. Phys. Rev. Lett. 2010, 104 (19), 197203 10.1103/PhysRevLett.104.197203. 20866995

[ref64] Sebastian T.; Bracher T.; Pirro P.; Serga A. A.; Hillebrands B.; Kubota T.; Naganuma H.; Oogane M.; Ando Y. Nonlinear Emission of Spin-Wave Caustics from an Edge Mode of a Microstructured Co2Mn0.6Fe0.4Si Waveguide. Phys. Rev. Lett. 2013, 110 (6), 067201 10.1103/PhysRevLett.110.067201. 23432296

[ref65] Gallardo R. A.; Alvarado-Seguel P.; Kakay A.; Lindner J.; Landeros P. Spin-wave focusing induced by dipole-dipole interaction in synthetic antiferromagnets. Phys. Rev. B 2021, 104 (17), 174417 10.1103/PhysRevB.104.174417.

[ref66] Sheka D. D.; Pylypovskyi O. V.; Landeros P.; Gaididei Y.; Kakay A.; Makarov D. Nonlocal chiral symmetry breaking in curvilinear magnetic shells. Communications Physics 2020, 3 (1), 128 10.1038/s42005-020-0387-2.

[ref67] Gallardo R. A.; Alvarado-Seguel P.; Landeros P. Unidirectional Chiral Magnonics in Cylindrical Synthetic Antiferromagnets. Physical Review Applied 2022, 18 (5), 054044 10.1103/PhysRevApplied.18.054044.

[ref68] Gallardo R A; Alvarado-Seguel P; Schneider T; Gonzalez-Fuentes C; Roldan-Molina A; Lenz K; Lindner J; Landeros P Spin-wave non-reciprocity in magnetization-graded ferromagnetic films. New J. Phys. 2019, 21, 033026 10.1088/1367-2630/ab0449.

