# Supplementary material for: Coherent Magnons with Giant Nonreciprocity at Nanoscale Wavelengths
Source: ACS Nano. 2024 Feb 5;18(7):5249–57. doi: 10.1021/acsnano.3c08390 (PMC10883124; doi:10.1021/acsnano.3c08390)
Supplement: Supplementary file 1 — nn3c08390_si_001.pdf [file nn3c08390_si_001.pdf]

Coherent magnons with giant non-reciprocity at nanoscale wavelengths
Supporting Information

Rodolfo Gallardo[1], Markus Weigand[2], Katrin Schultheiss[3], Attila Kakay[3], Roland Mattheis[4], Jörg Raabe[5], Gisela Schütz[6], Alina Deac[7], Jürgen Lindner[3], and Sebastian Wintz[2,6,*]

[1] Universidad Técnica Federico Santa María, 2390123 Valparaíso, Chile
[2] Helmholtz-Zentrum Berlin, 12489 Berlin, Germany
[3] Helmholtz-Zentrum Dresden-Rossendorf, Insitute of Ion Beam Physics and Materials Research, 01328 Dresden, Germany
[4] Leibniz Institut für Photonische Technologien. 07745 Jena, Germany
[5] Paul Scherrer Institut, 5232 Villigen PSI, Switzerland
[6] Max-Planck-Institut für Intelligente Systeme, 70569 Stuttgart, Germany
[7] Helmholtz-Zentrum Dresden-Rossendorf, Dresden High Magnetic Field Laboratory, 01328 Dresden, Germany
[*] email: wintz@is.mpg.de

(1) Static domain configurations and antiferromagnetic interlayer coupling

As resonant x-ray absorption is employed for the magnetic STXM imaging, the two different ferromagnetic layers of the Co/Ru/NiFe disk can be imaged individually, *i.e.* in a layer-resolving way. Figure S1 shows such an example of STXM images of the disk investigted with in-plane magnetic sensitivity for the Co layer (Co L_3 edge) (a) and the NiFe layer (Fe L_3 edge) (b). To a general extend, the sample is in a coupled vortex state with opposite in-plane vorticities between the two layers (as indicated by the orange arrows). However, there are some irregularities in the vortex domain pattern, and in particular at the lateral bottom right edge a second vortex core is present, congruently in both layers. While all general magnetic features exist in both layers, the Co layer exhibits an additional magnetic ripple structure (not to be confused with the scanning noise features in the NiFe image). From these results it can be concluded that the interlayer exchange coupling between the two ferromagnetic layers, mediated by the Ru layer, is generally antiferromagnetic. Note that for the main part of the sample, the vorticity combination is of opposite handedness than that discussed in the main manuscript. Therefore, in this region the slow spin-wave branch is propagating outwards whereas the fast branch is propagating inwards [*cf.* (2), Supporting Movie M5].

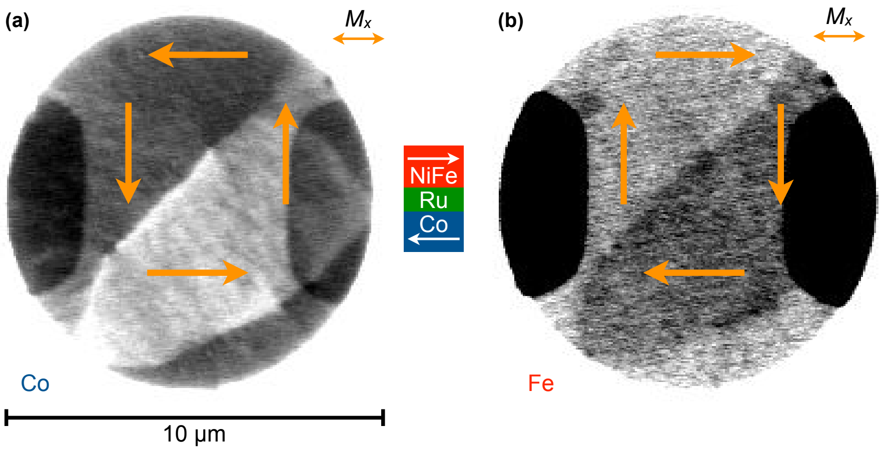

Figure S1: Layer-resolved STXM images of the Co/Ru/NiFe disk with in-plane magnetic sensitivity. (a) Co-layer, (b) NiFe layer, magnetic orientation as indicated by the orange arrows. Measurement data also shown in Ref. [47] of the main text.

(2) Supporting TR-STXM movies

Selected time-resolved scanning transmission x-ray microscopy measurements are provided as movies (**M1-M5**). These movies show contrast at different absorption edges (Fe and Co) and for different mounting geometries (0deg: pure perependicular magnetic contrast, 30deg: mixed perpendicular and lateral magnetic contrast along the *x*-axis), with scan sizes and resolutions on the micrometer and nanometer scale, respectively. The samples are excited by alternating currents at GHz frequencies, corresponding to ns periods, and their periodic response is recorded at time steps on the sub-100 ps scale. All these parameters are given for each movie by means of the file name as defined below:

***M*#_AbsorptionEdge_Geometry_ScanSize_ScanStep_Frequency_Period_TimeStep*

The different movie panels show - from left to right - : absolute absorption/magnetic contrast *(abs)* - normalized magnetic contrast (temporal changes) *(norm)* - blurred normalized contrast (only M1 and M2) *(normblur)*:

M1_Fe_0deg_10.5um2_28nm _3.57GHz_0.28ns_40ps_abs-norm-normblur
M2_Co_30deg_10um2_67nm_1.11GHz_0.90ns_39ps_abs-norm-normblur
M3_Fe_0deg_10.5um2_50nm_1.07GHz_0.93ns_133ps_abs-norm
M4_Co_30deg_2um2_20nm_2.07GHz_0.48ns_21ps_abs-norm
M5_Co_0deg_10um2_80nm_1.11GHz_0.90ns_39ps_abs-norm

All movies show 10 repetitions of the observation period for a better visibility of the dynamic effects. Besides movies M1 and M2 which were introduced in the main text, movies M3-M5 highlight additional aspects of the non-reciprocal spin waves discussed. Movie M3 shows the response of the sample to a very similar excitation as for movie M2, yet with pure perpendicular magnetic sensitivity. Here, the short wavelength branch becomes more evident than in movie M2, yet the fast branch can hardly be seen as a result of the strongly elliptical precession in the long wavelength limit. Movie M4 provides a magnified view on the high-amplitude inwards propagating short-wavelength branch of spin waves at 2 GHz frequency. Movie M5 displays the response of the sample at a state of reversed in-plane vorticities to an excitation frequency of 1.1 GHz. While the long-wavelength branch is not noticeable in this movie as for movie M3, here the short wavelength branch corresponds to spin waves propagating outwards from the center [*cf.* SI (1)]

(3) Acoustic and optic spin-wave modes

Besides the experimentally observed acoustic spin-wave mode with in-phase perpendicular dynamic magnetization components between the two layers, there is also an optic collective spin-wave mode with antiphase perpendicular dynamic magnetization components. Figure S2 displays the calculated spin-wave dispersion for both the acoustic and optic mode of collective spin waves in an antiparallel magnetic bilayer in the Damon-Eshbach geometry. The latter optic mode resides in principle at higher frequencies and it exhibits an inverted non-reciprocity (with respect to sgn k) yet of much smaller magnitude ($\lambda^+/\lambda^- < 1.5$) compared to the acoustic mode. Panels (b-e) illustrate the origin of the non-reciprocity discussed. Neglecting interlayer exchange coupling, there are different magnetostatic interactions for both the dynamic perpendicular and dynamic in-plane magnetization. While the perpendicular field/magnetization interaction is dominant (acoustic vs. optic mode), the fields from the dynamic in-plane component can either enhance or reduce the magnetostatic coupling energy/frequency (non-reciprocity).

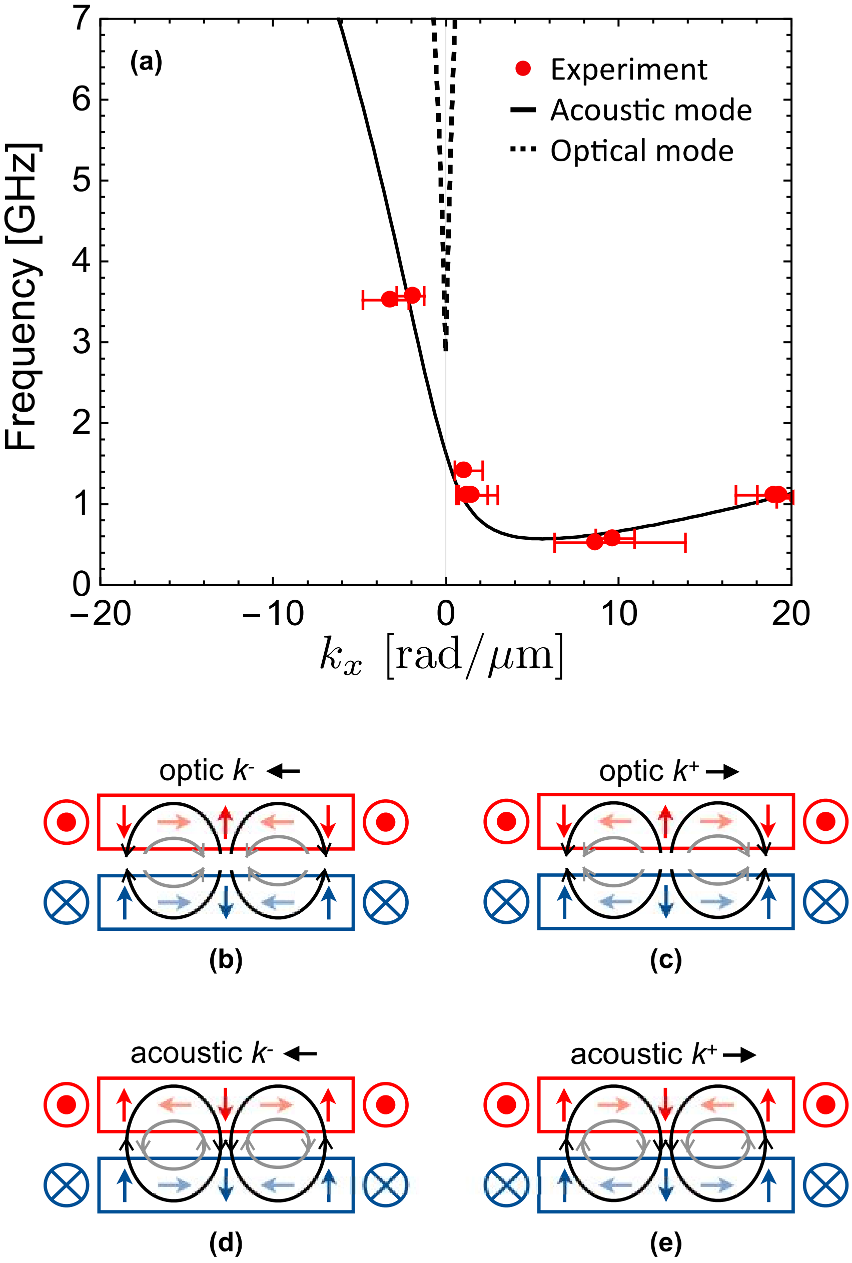

Figure S2: Non-reciprocity of optic and acoustic modes. (a) Calculated spin-wave dispersion relation for the acoustic (black solid line) and optic mode (black dashed line) with their non-reciprocal branches. (b-e) Schematics of the field interaction of the dynamic magnetization during the spin-wave dynamics with the equillibrium magnetization perpendicular to the paper plane as indicated aside the two layers. The magnetostatic energy incrases from (e) via (d) (acoustic), to (b) and (c) as a result of dipolar interactions between both the perpendicular and lateral dynamic magnetization components (black and grey arrows, respectively).

(4) Effect of the magnetic parameters on the spin-wave dispersion relation

In order to illustrate the effect of the magnetic parameters on the spin-wave dispersion relation, additional calculations were performed varying the following parameters with respect to the standard values individually by ± 20 %: g, J_L, d_{Ru}, d_{Co}, d_{NiFe}, A_{Co}, A_{NiFe}, M_{Co}, M_{NiFe}, $K_{u,Co}$, $K_{u,NiFe}$. The results are shown in Figure S3 for a k-range of -10 to +70 rad/µm. From these calculations, the following trends can be extracted for the asymmetric k-range considered here: As expected, the frequency scales with the g factor. A higher Ru interlayer thickness (dRu) mainly increases the slope of the slow branch which also follows from a negative interlayer exchange coupling (JL) with increased magnitude. A higher thickness of the ferromagnetic layers increases the slope of the fast branch and decreases the frequency of the dispersion minimum, with the effects being more pronounced for the NiFe layer. Higher exchange constants increase the slope of the slow branch. Higher saturation magnetizations decrease the slope of the slow branch and, in case of NiFe, also increases the slope of the fast branch and decreases the minimum frequency. Higher anisotropy constants increase the frequency at the dispersion minimum. Overall, there is no highly sensitive dependence of the general dispersion characteristics of the antiparallel bilayer on the specific magnetic properties.

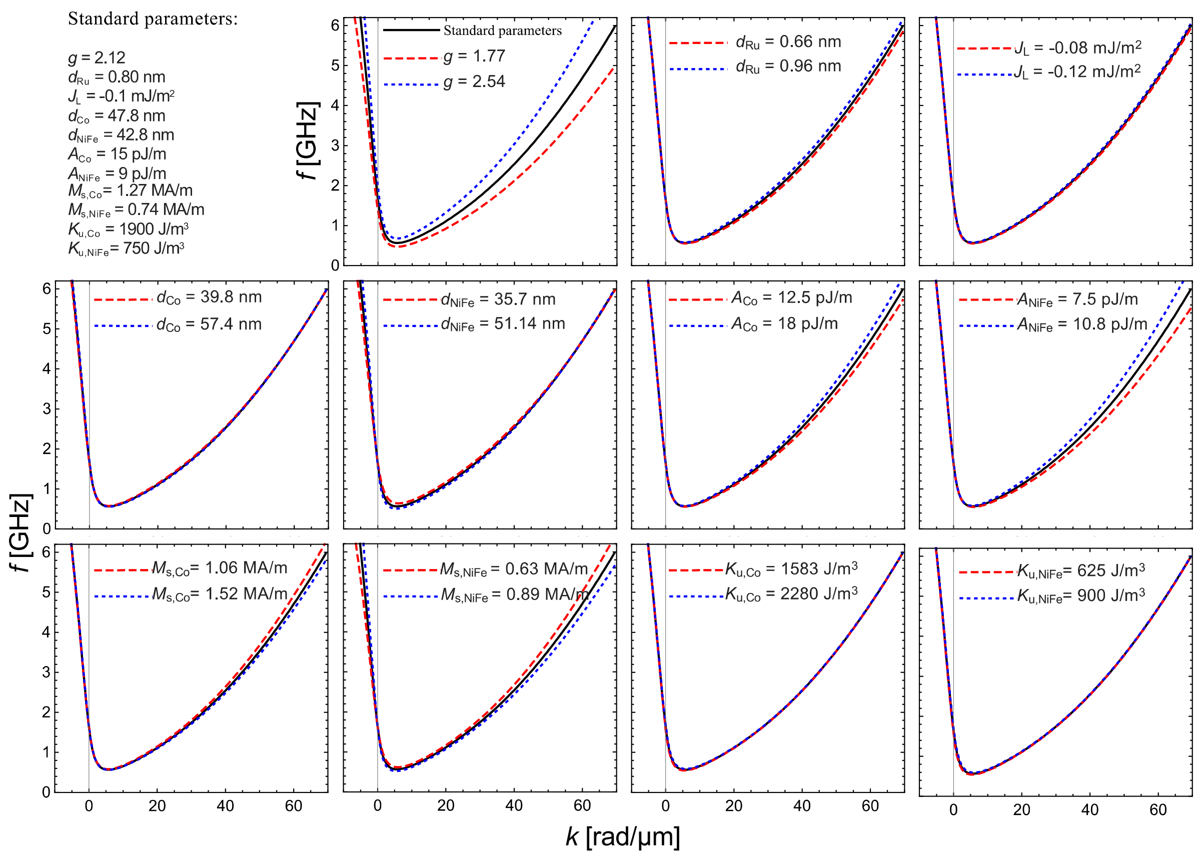

Figure S3: Calculation of the spin-wave dispersion relation for varied magnetic parameters.

(5) Higher order spin-wave modes and bands

Figure 4(b) of the main text illustrates the lowest energy spin-wave dispersion relation for all lateral k-directions with respect to the equilibrium magnetizations, aligned with the easy axis of intrinsic anisotropy. The graph reveals a strong dependence of the dispersion on the orientation of the lateral wavevector $\boldsymbol{k}_{xy}$, yet this dependence is symmetric with respect to the k_x axis. For small $|\boldsymbol{k}|$ values below 10 rad/µm, the dispersion corresponds to the fundamental acoustic spin-wave mode of the antiparallel magnetic bilayer that -in the Damon-Eshbach geometry ($\boldsymbol{M}_{eq} \perp \boldsymbol{k}$)- we experimentally observe as shown in Figures 3 and S2.

Besides the acoustic and optic fundamental mode of the antiparallel magnetic bilayer, there are also modes of higher perpendicular order at higher frequencies, bearing precessional nodes over the thickness of the individual ferromagnetic films. Figure S3 shows the calculated spin-wave dispersion relations of the two lowest of such higher order modes for the principal orientations Damon-Eshbach ($\boldsymbol{M}_{eq} \perp \boldsymbol{k}$) S4(a) and backward-volume ($\boldsymbol{M}_{eq} || \boldsymbol{k}$) S4(b) together with the acoustic and optic mode. Note that in the backward-volume geometry all four modes are well separated and they do not cross each other for the k-range given. Here, the spin-wave *modes* (indicated by line type and defined by the spin-wave nodal character) are identical with the spin-wave *bands* (indicated by color and corresponding to a continuous dispersion function). This situation changes when considering the Damon-Eshbach geometry, where different spin-wave modes intersect. Namely the fast branch of the acoustic mode and both branches of the optic mode cross the two lowest higher order modes. This modal crossing, however, only occurs with respect to the spin-wave nodal character. The continous dispersion functions (referred to as spin-wave *bands*) in fact do not intersect but exhibit avoided crossings instead.

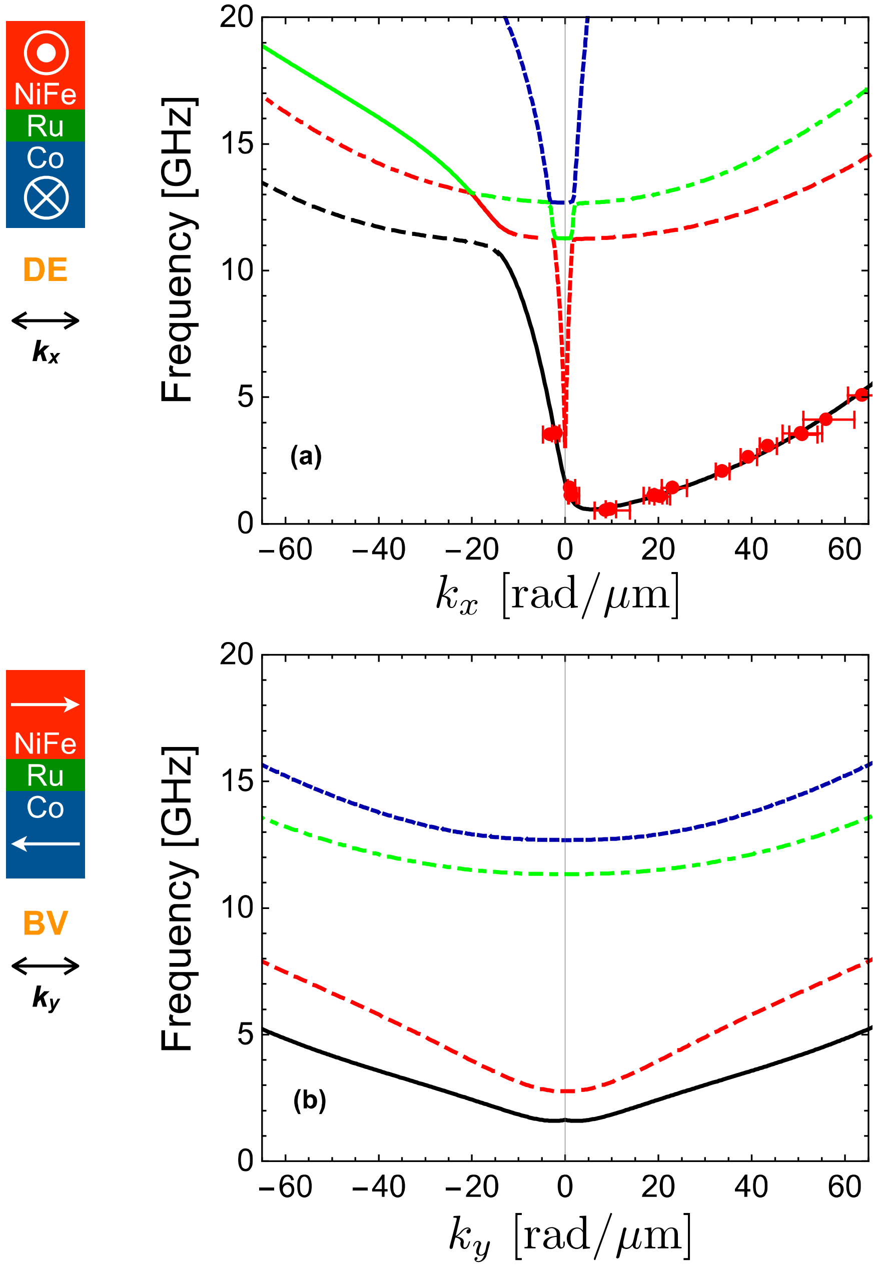

Figure S4: Calculated spin-wave dispersion relations *f*(*k*) for the four lowest spin-wave modes and bands. (a) Damon-Eshbach geometry and (b) Backward-volume geometry. Crossing spin-wave modes are indicated by line type, anti-crossing spin-wave bands are indicated by color.

(6) Identifying points of zero curvature and the lines these points form in the isofrequency contours of the spin-wave dispersion relation *f (k$_x$,k$_y$)*

$f(k_x,k_y)$ is a function $f{:}\mathbb{R}^2{\to}\mathbb{R}$. Let $\tau{\to}\varphi(\tau)$ be a parametrization of a curve connecting the points of zero curvature of isolines. Let τ_0 and $\varphi(\tau_0)$ indicate an arbitrary point on that curve. Then, there is a unique isoline going through that point. Let $\varepsilon{\to}\psi(\varepsilon)$ be a parametrization of that isoline, with $\psi(0) = \varphi(\tau_0)$.

Now, following the isoline, we can state:

$$f(\psi(\varepsilon)) = const. \tag{S1}$$

and thus,

$$0 = \frac{d^2}{d\varepsilon^2} f(\psi(\varepsilon)) = \frac{d}{d\varepsilon}\left(\frac{\partial f}{\partial x}\frac{d\psi_x}{d\varepsilon} + \frac{\partial f}{\partial y}\frac{d\psi_y}{d\varepsilon}\right) = \begin{pmatrix}\frac{d\psi_x}{d\varepsilon} & \frac{d\psi_y}{d\varepsilon}\end{pmatrix}\begin{pmatrix}\frac{\partial^2 f}{\partial x^2} & \frac{\partial^2 f}{\partial x \partial y} \\ \frac{\partial^2 f}{\partial x \partial y} & \frac{\partial^2 f}{\partial y^2}\end{pmatrix}\begin{pmatrix}\frac{d\psi_x}{d\varepsilon} \\ \frac{d\psi_y}{d\varepsilon}\end{pmatrix} + \begin{pmatrix}\frac{\partial f}{\partial x} \\ \frac{\partial f}{\partial y}\end{pmatrix} \cdot \frac{d^2}{d\varepsilon^2}\psi. \tag{S2}$$

Now, since we are on the curve φ, at $\varepsilon = 0$ the last term of the above equation vanishes. Thus, we have

$$\begin{pmatrix}\frac{d\psi_x}{d\varepsilon} & \frac{d\psi_y}{d\varepsilon}\end{pmatrix}\begin{pmatrix}\frac{\partial^2 f}{\partial x^2} & \frac{\partial^2 f}{\partial x \partial y} \\ \frac{\partial^2 f}{\partial x \partial y} & \frac{\partial^2 f}{\partial y^2}\end{pmatrix}\begin{pmatrix}\frac{d\psi_x}{d\varepsilon} \\ \frac{d\psi_y}{d\varepsilon}\end{pmatrix} = 0. \tag{S3}$$

Now, $\psi(\varepsilon)$ can be chosen such that

$$\frac{d\psi}{d\varepsilon}(0) = \begin{pmatrix}0 & 1 \\ -1 & 0\end{pmatrix}\nabla f(\varphi(\tau_0)). \tag{S4}$$

Thus, we can state that the function g defined as

$$g = \nabla f^T \begin{pmatrix} \frac{\partial^2 f}{\partial y^2} & -\frac{\partial^2 f}{\partial x \partial y} \\ -\frac{\partial^2 f}{\partial x \partial y} & \frac{\partial^2 f}{\partial x^2} \end{pmatrix} \nabla f \tag{S5}$$

vanishes at the points of zero curvature.
